# A surface acoustic wave-driven micropump for particle uptake investigation under physiological flow conditions in very small volumes

**DOI:** 10.3762/bjnano.6.41

**Published:** 2015-02-09

**Authors:** Florian G Strobl, Dominik Breyer, Phillip Link, Adriano A Torrano, Christoph Bräuchle, Matthias F Schneider, Achim Wixforth

**Affiliations:** 1 Lehrstuhl für Experimentalphysik I, Universität Augsburg, 86159 Augsburg, Germany; 2 Nanosystems Initiative Munich NIM, Schellingstr. 4, 80799 Munich, Germany; 3 Department of Chemistry and Center for NanoScience (CeNS), University of Munich (LMU), 81377 Munich, Germany; 4 Department for Mechanical Engineering, Boston University, Boston, MA 02215, USA

**Keywords:** acoustic streaming, cellular uptake, flow, nanoparticles, sedimentation, shear, surface acoustic wave (SAW)

## Abstract

Static conditions represent an important shortcoming of many in vitro experiments on the cellular uptake of nanoparticles. Here, we present a versatile microfluidic device based on acoustic streaming induced by surface acoustic waves (SAWs). The device offers a convenient method for introducing fluid motion in standard cell culture chambers and for mimicking capillary blood flow. We show that shear rates over the whole physiological range in sample volumes as small as 200 μL can be achieved. A precise characterization method for the induced flow profile is presented and the influence of flow on the uptake of Pt-decorated CeO_2_ particles by endothelial cells (HMEC-1) is demonstrated. Under physiological flow conditions the particle uptake rates for this system are significantly lower than at low shear conditions. This underlines the vital importance of the fluidic environment for cellular uptake mechanisms.

## Introduction

**Figure 1:**
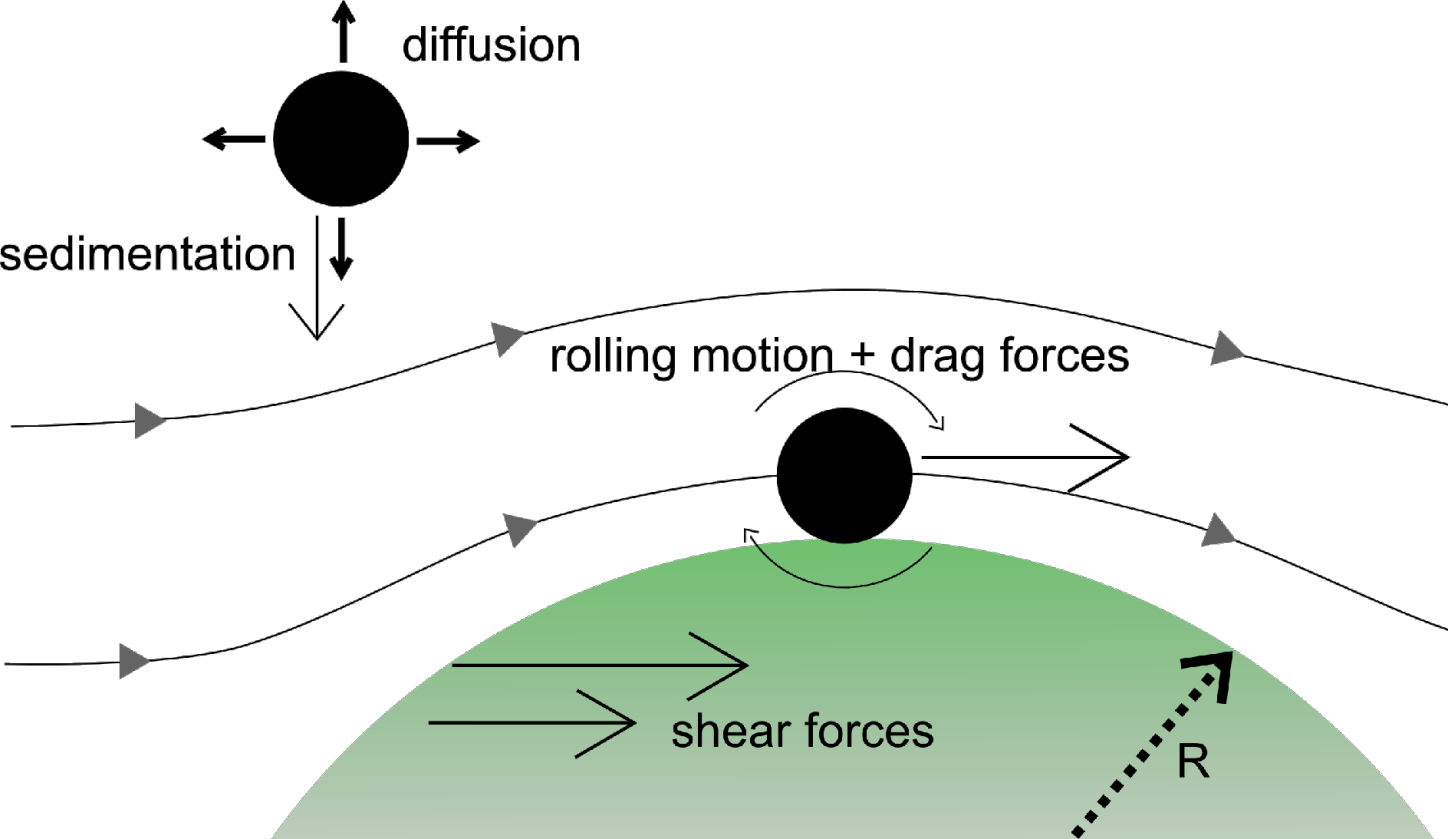
The uptake of particles by a cell is influenced by different factors: diffusion and sedimentation will limit the maximum particle delivery for static conditions. Under flow, these factors become less important, but shear forces acting on the cell and the particles will influence the uptake mechanisms.

Most in vitro experiments performed to investigate nanoparticle–cell interactions are done under static flow conditions, with adherent cells residing at the bottom of a culture slide. This can be an important flaw when it comes to the quantitative interpretation of experimental data [1]. Providing that the cellular uptake mechanisms are fast enough, the particle uptake rate at a given particle concentration in the medium, *C*_m_ , will be limited by the particle motion and the re-supply in the medium. For small particles, diffusion will dominate the delivery rate d*N*/d*t*. For the sake of simplicity, assume the observed cell being a half-sphere with radius R as depicted in Figure 1.

If the influence of gravity can be neglected, the particle delivery rate at equilibrium can then be derived from Fick’s law [2]:

[1]
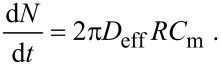


Here *D*_eff_ denotes an effective diffusion constant.

In an in vitro experiment without fluid motion, however, also sedimentation will contribute to the delivery rate. For very big particles or, probably more relevant, for particle agglomerates, the delivery rate will be determined by the sinking velocity, which is governed by the Stokes equation:

[2]
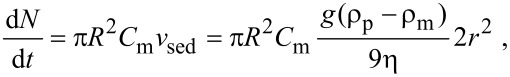


where *v*_sed_ is the sedimentation velocity and ρ_p_ and ρ_m_ are the densities of particle and medium, respectively.

Under realistic conditions, there will be, after some time, an equilibrium between sedimentation and back-diffusion and, hence, the cell will be exposed to an elevated local particle concentration *C*_loc_ > *C*_m_. Ignoring these effects can in fact lead to misinterpretation of experimental data, especially to an overestimation of the impact of big particles or agglomerates.

Furthermore, particles on a cell surface under shear are subject to drag and torsion forces [3]. For spherical particles in the nano-regime it can be easily derived from the Stokes equation that these drag forces are typically of the order of a few piconewtons or below, i.e, one or more orders of magnitude weaker than typical receptor–ligand binding strengths [4]. Nevertheless, in the case of weak unspecific particle adhesion, these forces can be strong enough to induce a rolling motion over the cell, which in turn significantly reduces the mean contact time between particle and cell and, hence, the uptake probability. Assuming a rolling particle with radius *r* = 50 nm and following its center streamline at a shear rate of 2000 s^−1^, its rolling velocity will be of the order of 100 μm/s. Relevant disruption forces for specific bindings can be achieved for large particle agglomerates, since the drag force scales with *r*^2^. An agglomerate with an hydrodynamic radius of *r* = 500 nm at a shear rate of 2000 s^−1^, for instance, experiences a force of approximately 77 pN [5].

Finally, in many studies, endothelial cells are of special interest due to their outstanding role regarding the distribution of nanoparticles by the vascular system. Hence, another important issue is that a static medium is certainly not a physiological surrounding for these cells, which are exposed in vivo to shear rates of up to 3000 s^−1^ [6]. It was recently shown that the glycocalix of endothelial cells is substantially reorganized under shear [7] and several effects of shear stress on cellular uptake mechanisms have been reported [8–10].

One solution to the aforementioned problems is to perform experiments under realistic flow conditions. In the following, we introduce a novel device for inducing high shear rates at the bottom of an arbitrary cell culture chamber. The device is based on SAW-driven acoustic streaming. Many different applications of this effect in the area of microfluidics and life science have been developed so far (see reviews [11–12]). In the past, our lab already introduced SAW devices for quantifying cell association of targeted particles [13] and cell adhesion on implant materials [14]. One of the advantages of SAW-driven systems is their applicability to very small samples without having to deal with any dead volume. Thus, the demand for sample material is extremely low and the small surfaces in general reduce the risk of sample contamination. Moreover, the devices are typically very robust and inexpensive to produce. However, the shear rates that were reported so far for “conventional” SAW-driven microfluidic devices are usually one or two orders of magnitude below the typical shear rates of the capillary system and therefore not suitable for mimicking capillary blood flow. High input power to the SAW generator is no solution to that problem since dissipation would heat the sample. However, as we will show, the application of focusing interdigital transducers (FIDTs) in an L-shape configuration allows for shear rates of up to 4000 s^−1^ without significant sample heating.

## Results and Discussion

### Microfluidic setup

**Figure 2:**
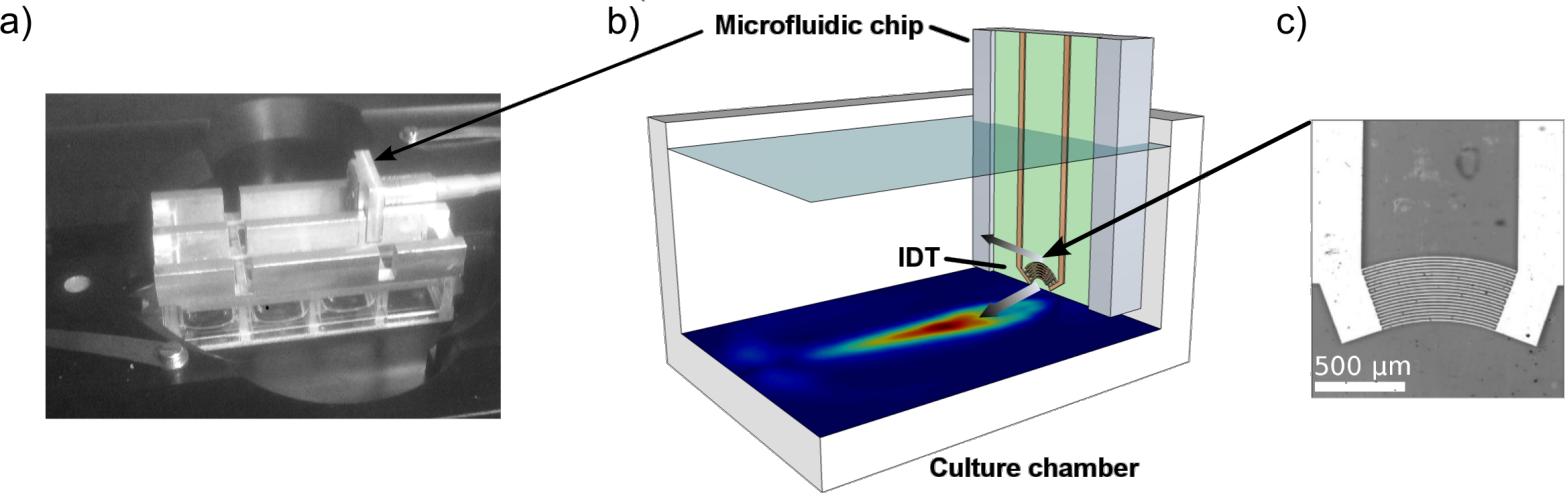
a) Photograph of the sample system on a microscope stage. The chip is mounted onto a culture slide by a metal frame. b) Sketch of the chip. A focusing interdigital transducer induces acoustic streaming with the main flow component pointing downwards, incident at the bottom in an angle of approximately 23° and inducing shear stress on the surface. c) Micrograph of the FIDT.

Our device is illustrated in Figure 2. In short, applying a high-frequency voltage to an interdigital transducer (IDT) on a piezoelectric substrate induces Rayleigh-mode SAWs. The latter couple into the fluid medium and excite longitudinal pressure waves. As such high-frequency pressure waves are attenuated on short distances, acoustic radiation pressure is generated which eventually induces fluid motion [15].

The device can in principle be attached to an arbitrary culture chamber. Its design offers a possibility to perform experiments under flow without changing the culture procedures or environments as compared to the static case. In the sample setup shown in Figure 2b the SAW-micropump is mounted on top of a Nunc^TM^ Lab-Tek^TM^ chamber slide through an aluminum frame. Instead of a standard, straight IDT, a circular arc focusing IDT (FIDT) is employed. (For technical details see Experimental section.) The steady body force and hence the streaming velocity scales quadratically with the amplitude of the sound waves [16]. As a consequence, focusing the acoustic energy results in a non-linear increase of the energy stored in the fluid motion. Singh et al. [17] analyze the effect of focusing on the streaming efficiency numerically and report significant advantages of focusing over standard, linear IDTs. Several groups used the advantages of focusing transducer devices for microfluidic applications before [18–21]. Unpublished data from experiments in our group indicate that the integrated kinetic energy of the streaming profile can be several orders of magnitude larger for optimized FIDTs than for standard IDTs at similar experimental conditions. This aspect is subject to ongoing work.

SAW-driven microfluidic pumps are usually designed as planar structures that require either a sample observation through the piezoelectric substrate or a coupling of the acoustic power into the sample chamber. This means a loss of either optical quality or energetic efficiency. The L-shape structure of this setup allows for the application of standard cell imaging slides while directly coupling the sound waves into the fluid medium. Additionally, the angle of incidence of the generated fluid jet in this design is equal to the Rayleigh angle of about 23° and thus favors the desired generation of shear force in the x–y plane without the need for a confinement of the flow profile. Moreover, the piezoelectric substrate has no direct connection to the bottom of the cell chamber and can transfer heat to the outer metal frame. This reduces the heat input into the sample chamber. Measuring the bulk temperature during experiments with living cells (systems at 37 °C) over 1 h showed no significant heating due to dissipation at the applied input power of *P*_SAW_ ≈ 19 dBm.

### Characterization of the flow pattern

**Figure 3:**
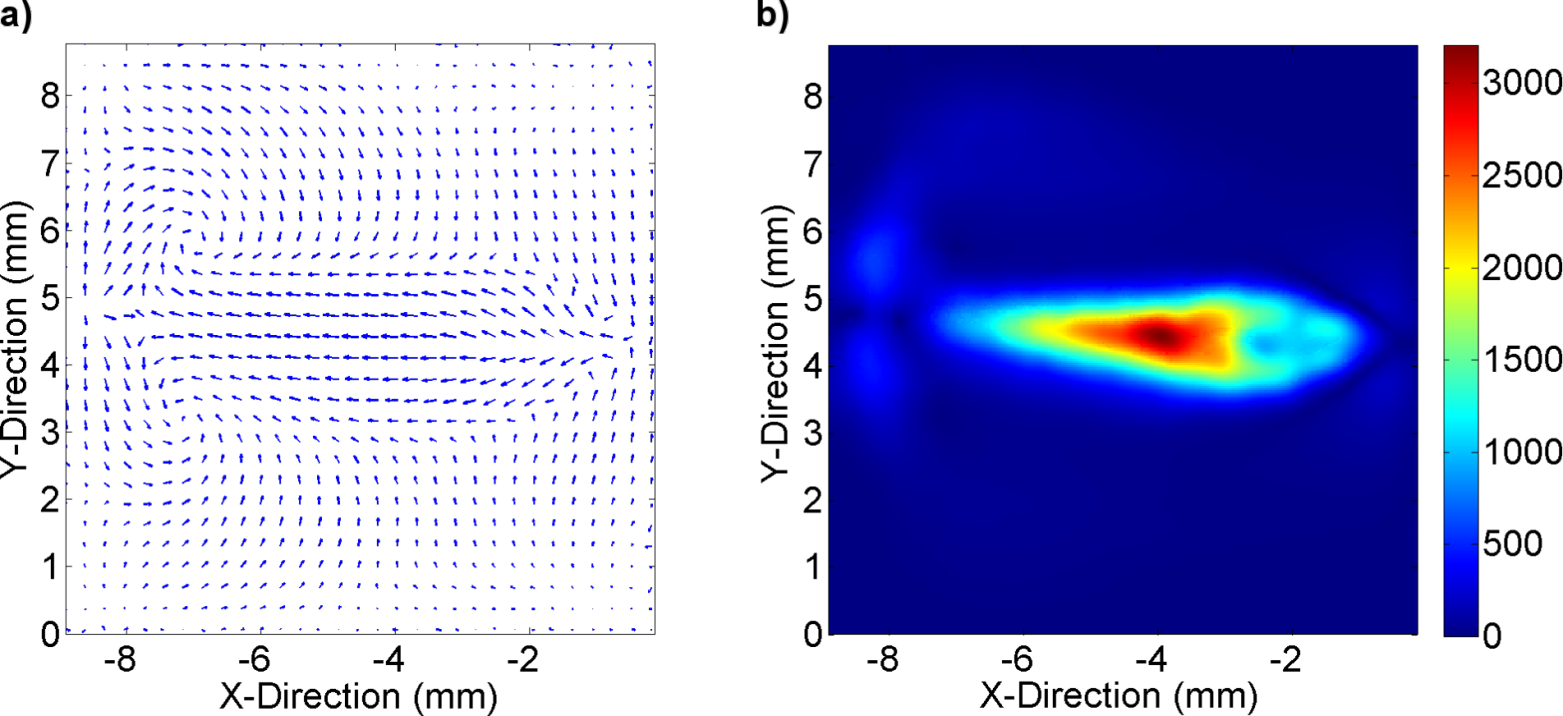
a) The velocity profile at a distance of *z* = 10 μm from the chamber bottom. The vector length scales with the normalized logarithmic velocity. b) The color code indicates the respective bottom shear rate in s^−1^.

The characterization of the SAW-induced velocity field is done by particle image velocimetry (PIV). For improving the resolution while being able to capture the whole chamber the method is performed using a scanning approach (SPIV) that is described in the Experimental section. This characterization procedure is necessary for each combination of IDT-parameters and chamber geometry, since both will change the flow profile. For our purposes, the near bottom shear rate


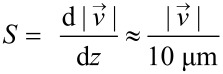


is the most interesting value. Figure 3 illustrates the bottom flow conditions at an effective input power of *P*_SAW_ ≈ 19 dBm.

Within the jet region, the whole range of physiological relevant shear rates, i.e., from 100 s^−1^ up to 3000 s^−1^ is covered. By observing cells within different areas of interest, several shear rates can be monitored in the same experiment. The peripheral regions exhibit only very little motion and can serve as reference for near-zero shear, although they still experience some medium exchange. It should be mentioned that even though the main streamline of the FIDT points towards the chamber bottom, some streaming in upper direction is generated, too. This represents an advantageous side effect, since both streams meet in the central region of the chamber and generate streamline folding (see also Figure 2b), leading to an efficient mixing of the medium, as reported earlier [22].

### Particle uptake under flow

**Figure 4:**
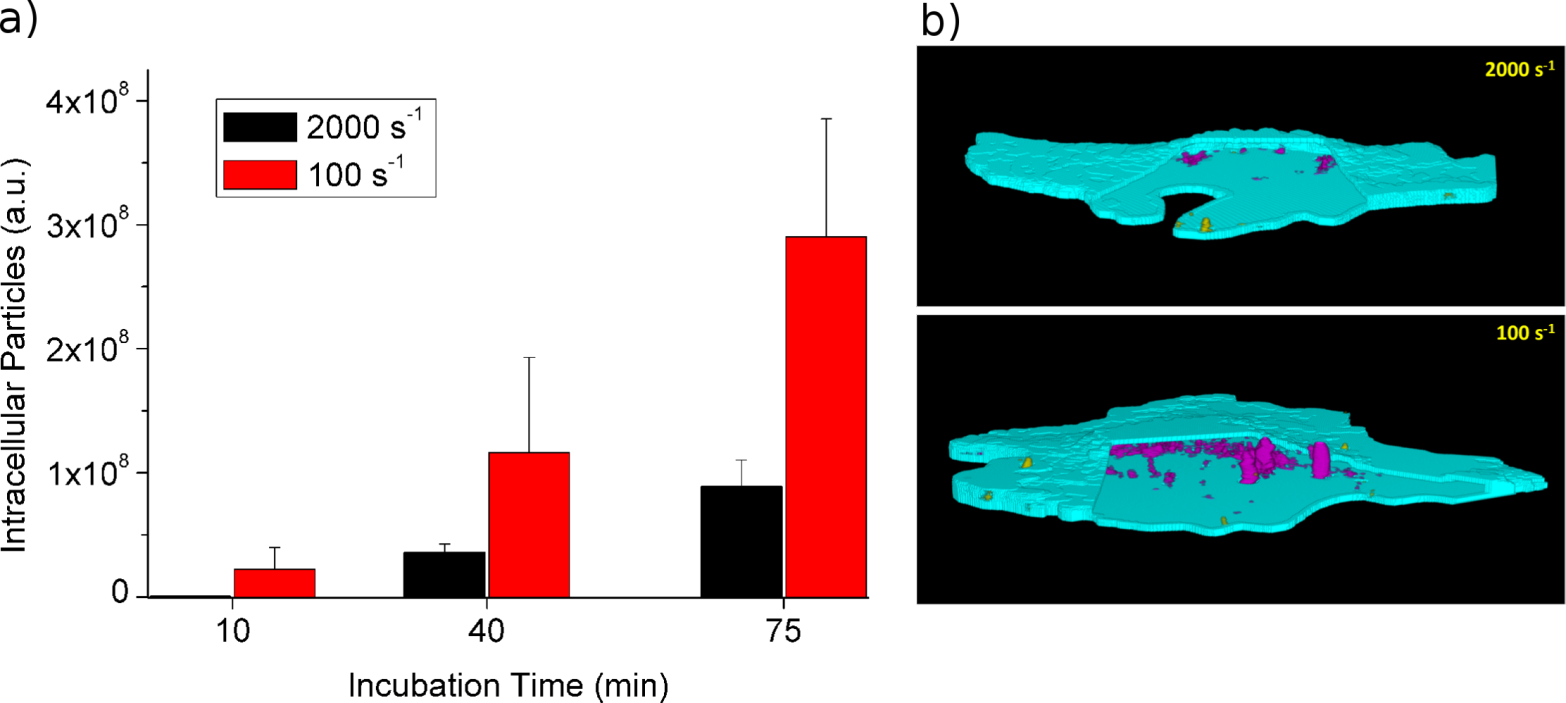
a) Total fluorescence of internalized particles (*d* = 50 nm) at different shear rates. b) Two representative cells, analyzed with *Particle in Cell-3D*. Internalized particles appear in pink, membrane associated particles in yellow.

In order to show the relevance of physiological shear conditions for the uptake of nanoparticles in cells and to prove the applicability of our system, we examined the uptake of Pt-decorated CeO_2_ particles (*d* = 50 nm) by HMEC-1 cells. The cells were incubated with cell medium containing the nanoparticles and under flow conditions characterized as described above. The amount of particles taken up by the cells was then analyzed at different incubation times. Figure 4 compares the results for two different regions of interest (ROIs) with shear rates of 100 s^−1^ and 2000 s^−1^, respectively.

For both ROIs the uptake develops approximately linearly in time, but a clear difference in the uptake efficiency at different shear rates can be seen. At a moderate shear rate (100 s^−1^), after 75 min the uptake is about seven times higher than at a high (physiological) shear rate of 2000 s^−1^. Sedimentation and long range diffusion should not be relevant here, since under both shear conditions the flow provides continuous medium exchange. Hence, the observed effects are mainly related to the uptake process itself and its dependence on flow strength and particle-cell contact time. This interesting aspect is presently under investigation.

## Conclusion

In summary, we have shown that fluidic conditions can be of vital importance for cellular uptake processes. This aspect is especially important when examining endothelial cells and/or the uptake of particles where sedimentation could be an issue and should not be omitted in respective experiments. We introduced a setup for SAW-induced pumping that can be used for mimicking physiological flow conditions in cell experiments and has several advantages over state-of-the-art solutions, for instance, shear rates over the whole physiological range and applicability with arbitrary standard culture slides. Scanning particle imaging velocimetry has shown to be suitable for generating near bottom shear rate maps that can easily be correlated to biological results.

Our experimental data on the uptake behavior of HMEC-1 cells show that the particle uptake under physiological high shear conditions is much lower than at low shear rates. This underlines the high importance of fluidic conditions for cellular particle uptake and demonstrates that disregarding these aspects can lead to severe misinterpretation of experimental data.

## Experimental

### Microfluidic chip

The general design of the setup is described in the Results and Discussion section. Here, we want to give the technical parameters of the used FIDT (see also Figure 2c). The structure is circular focused with a focus distance of 750 μm from the electrode and an opening angle of 40°. The chip consists of 20 fingers with an interdigital distance of 15 μm. The structure is fabricated by thermal evaporation of 50 nm gold on a LiNbO_3_ substrate (128°–Y–cut). For the sake of chemical and electrical isolation, the whole chip was covered with a 200 nm thick silicon oxide layer by thermal evaporation of SiO. The micropump was driven by an effective RF power of *P*_SAW_ ≈ 19 dBm at its resonance frequency of 126 MHz.

### Flow characterization

The SAW-induced flow pattern is characterized by scanning particle imaging velocimetry (SPIV). The flow is made visible by 3 μm polystyrene beads and the chamber is scanned in several x–y layers with position distances according to the field of view of the optical setup. With this approach the whole chamber volume can be imaged with sufficiently high magnification (and respective numerical aperture) to achieve a z-resolution that is adequate for a reliable analysis of the near bottom shear rates. The layer-to-layer distance for recording the shear rate map presented in Figure 3 is Δ*z* = 25 μm with the first layer at a height *z* = 10 μm above the bottom. At each position, 50 frames at a rate of 3000 fps are captured. A *MATlab* script based on the open source *PIVlab* toolkit [23–25] is applied to extract the three-dimensional velocity profile in following steps:

- Single videos are analyzed in a batch process by using *PIVlab* to determine the local velocity profiles.
- The results at single positions are stitched and missing data points are recovered by linear interpolation, ending up with layered x–y velocity profiles for the whole region of interest (ROI).
- If desired, z-velocities *v*_z_ can be extracted from the divergence 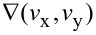 of the local field with appropriate boundary conditions.

### Uptake experiments

HMEC-1 cells were cultured as described in this journal elsewhere [26]. Five randomly chosen cells were analyzed at every ROI. Before starting the measurement, the culture medium was exchanged by medium containing Pt-decorated CeO_2_ particles with a concentration of 100 μg/mL. Cells and particles were then imaged with a spinning disc fluorescence microscope. Finally, the amount of internalized particles was determined for different incubation times employing the *Particle in Cell-3D* plugin for *ImageJ* that was presented elsewhere [26–27]. Synthesis and characterization of these particles are described elsewhere in this journal [28]).
